# Standardized Diagnostics Including PET-CT Imaging, Bilateral Tonsillectomy and Neck Dissection Followed by Risk-Adapted Post-Operative Treatment Favoring Radio-Chemotherapy Improve Survival of Neck Squamous Cell Carcinoma of Unknown Primary Patients

**DOI:** 10.3389/fonc.2021.682088

**Published:** 2021-05-07

**Authors:** Gunnar Wichmann, Maria Willner, Thomas Kuhnt, Regine Kluge, Tanja Gradistanac, Theresa Wald, Sandra Fest, Florian Lordick, Andreas Dietz, Susanne Wiegand, Veit Zebralla

**Affiliations:** ^1^ Department of Otorhinolaryngology, Head and Neck Surgery, University Hospital Leipzig, Leipzig, Germany; ^2^ Department of Radiation Oncology, University Hospital Leipzig, Leipzig, Germany; ^3^ Department of Nuclear Medicine, University Hospital Leipzig, Leipzig, Germany; ^4^ Department of Pathology, University Hospital Leipzig, Leipzig, Germany; ^5^ Department of Internal Medicine II, Division of Oncology, University Cancer Center Leipzig (UCCL), Leipzig University Medicine, Leipzig, Germany

**Keywords:** cancer of unknown primary (CUP), neck dissection (ND), extracapsular extension of neck nodes (ECE), cisplatin-based postoperative radio-chemotherapy, outcome research, head and neck cancer (HNC), head and neck squamous cell carcinoma (HNSCC)

## Abstract

**Background:**

About five to 10% of cancers in the head and neck region are neck squamous cell carcinoma of unknown primary (NSCCUP). Their diagnosis and treatment are challenging given the risk of missing occult tumors and potential relapse. Recently, we described human papillomavirus (HPV)-related NSCCUP-patients (NSCCUP-P) as a subgroup with superior survival. However, standardized diagnostic workup, novel diagnostic procedures, decision-making in the multidisciplinary tumor board (MDTB) and multimodal therapy including surgery and post-operative radio-chemotherapy (PORCT) may also improve survival.

**Methods:**

For assessing the impact of standardized diagnostic processes simultaneously established with the MDTB on outcome, we split our sample of 115 NSCCUP-P into two cohorts treated with curative intent from 1988 to 2006 (cohort 1; *n* = 53) and 2007 to 2018 (cohort 2; *n* = 62). We compared diagnostic processes and utilized treatment modalities applying Chi-square tests, and outcome by Kaplan–Meier plots and Cox regression.

**Results:**

In cohort 2, the standardized processes (regular use of [^18^F]-FDG-PET-CT imaging followed by examination under anesthesia, EUA, bilateral tonsillectomy and neck dissection, ND, at least of the affected site) improved detection of primaries (*P* = 0.026) mostly located in the oropharynx (*P* = 0.001). From 66.0 to 87.1% increased ND frequency (*P* = 0.007) increased the detection of extracapsular extension of neck nodes (ECE+) forcing risk factor-adapted treatment by increased utilization of cisplatin-based PORCT that improved 5-years progression-free and overall survival from 60.4 and 45.3 to 67.7% (*P* = 0.411) and 66.1% (*P* = 0.025).

**Conclusions:**

Standardized diagnostic workup followed by ND and risk-factor adapted therapy improves survival of NSCCUP-P.

## Introduction

The earliest description of cervical lymph node metastasis as the primal symptom of cancer by Hayes Martin dates back to 1944 (1). Today a subgroup of about 5% of head and neck cancer cases (2) and 3–5% of all human cancer cases (3) are diagnosed based on a lump in the neck (4) without obvious signs for a primary tumor and are designated neck squamous cell carcinoma of unknown primary (NSCCUP). Identification of occult primary tumors is challenging, as small flat lesions in anatomical complex structures of the oropharynx, oral cavity, hypopharynx and larynx may be invisible and impalpable (5, 6). Therefore, and as NCCN and ASCO guidelines recommend (7, 8), clinical examinations shall be accompanied by radiological imaging using ultrasound sonography and computed tomography (CT) or magnet-resonance imaging (MRI) upfront excision biopsies of potentially affected regions, as well as examination under anesthesia (EUA) involving bilateral tonsillectomy (9).

New imaging technologies combining [^18^F]-FDG positron-emission tomography (PET) and CT (PET-CT) or MRI (PET-MRI) facilitate detection of occult tumors and metastasis (10, 11). Therefore, ASCO recommends PET-CT/PET-MRI imaging followed by EUA and adapted treatment planning (8). However, to the best of our knowledge, literature showing facilitated diagnostic processes, decision-making for particular treatment leading to improved survival of NSCCUP-patients (NSCCUP-P) based on full implementation of these guidelines (7, 8) does not exist.

We established a multidisciplinary tumor board (MDTB) for discussing each individual case aiming on consented recommendations for the evidence-based probably best treatment option. We implemented all now recommended diagnostic procedures and risk factor-adapted treatment already in 2007. By comparing outcome in our cohort of NSCCUP-P before and after implementation of the MDTB and standardization of workup including risk factor-adapted treatment, we show the benefit from this sound approach.

## Materials and Methods

### Study Population and Patient Samples

**Figure 1:**
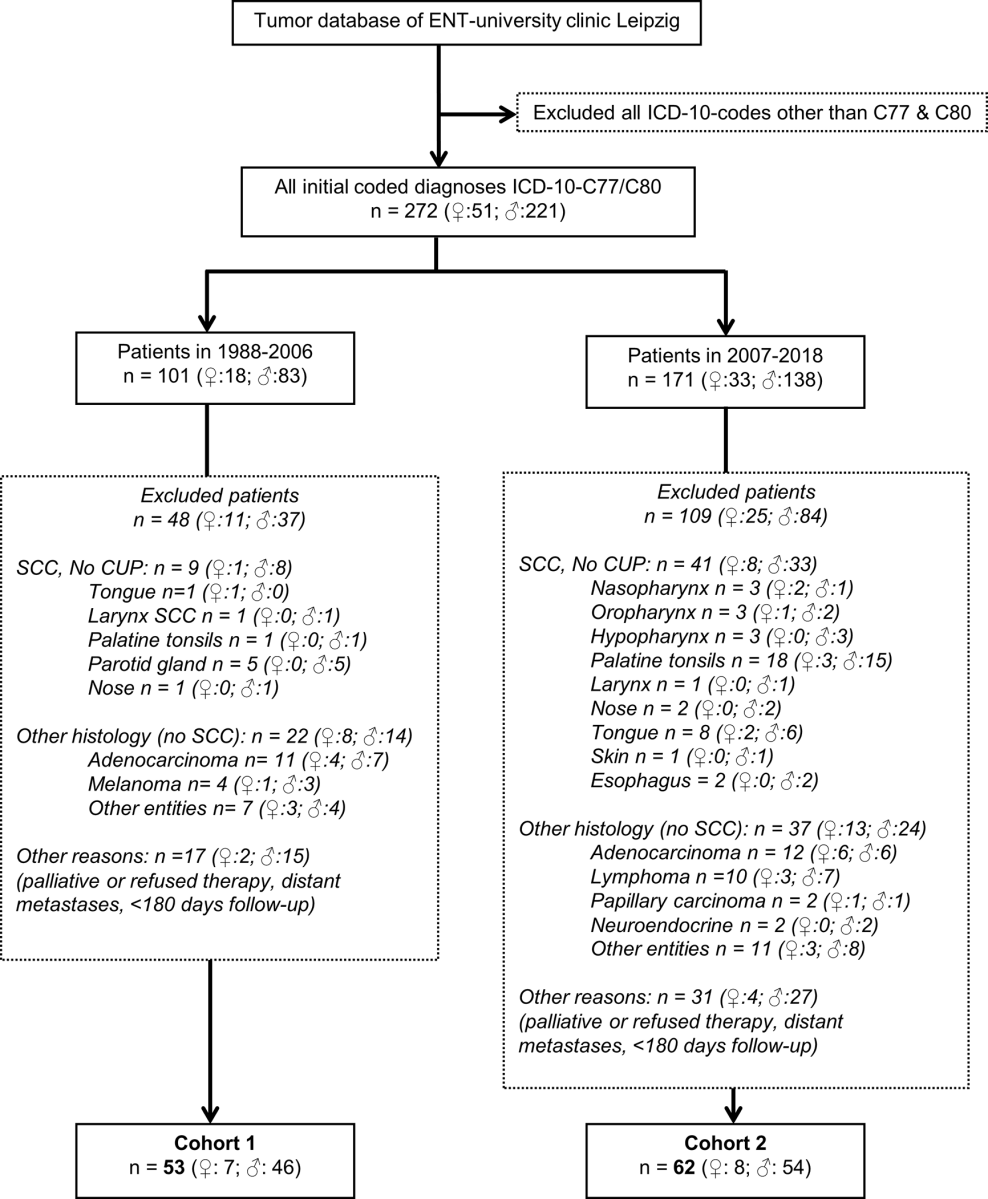
CONSORT diagram showing the selection criteria of neck squamous cell carcinoma of unknown primary (NSCCUP) patients of the two cohorts before and after standardization of diagnostic workup and therapy as well as the distribution of primary tumors detected leading to exclusion of initially suspect NSCCUP patients.

The study was conducted according to the guidelines of the Declaration of Helsinki, and approved by the Ethics Committee of the University Leipzig (votes 201-10-12072010 and 202-10-12072010). Patients diagnosed at our clinic between 1988 and 2018 with a lump in the neck without clinical sign of a primary tumor (ICD-10-C77 or ICD-10-C80) are included and underwent diverse diagnostics until establishing the MDTB and standardizing the diagnostic workflow. Finally, we analyzed 115 pathological confirmed NSCCUP-P out of 272 cases in two cohorts (**Figure 1**).

### Clinical Work-Up for NSCCUP-P

Clinical work-up for NSCCUP-P until 2006 (cohort 1) varied and included e.g. clinical examination, ultrasound sonography and other variable procedures (**Table 1**) before treatment. Since 2007 (cohort 2) clinical work-up was standardized and included, as recommended (7, 8), clinical examination, ultrasound sonography, contrast-enhanced CT, PET-CT/PET-MRI followed by EUA accompanied by taking multiple biopsies from the epipharynx, base of the tongue or lingual tonsillectomy plus bilateral tonsillectomy. Patient and tumor characteristics, diagnostic procedures, treatment and clinical follow-up were recorded in our Microsoft Access^®^ tumor database (TDB) and OncoFlow^®^ (12–14) (**Tables 1** **–** **3**).

**Table 1:** Clinical and epidemiological characteristics, diagnostic procedures and treatment of neck squamous cell carcinoma of unknown primary (NSCCUP) patients of cohorts 1 and 2.

Clinical and epidemiological characteristics		Total	Cohort 1 *n* (%)		Cohort 2 *n* (%)		*P* value^#^
Sex	female	15	7	(13.2)	8	(12.9)	0.961
	male	100	46	(86.8)	54	(87.1)	
Age at diagnosis	< 60 years	66	32	(60.4)	34	(54.8)	0.549
	≥ 60 years	49	21	(39.6)	28	(45.2)	
N-category TNM 2010	N1	17	8	(15.1)	9	(14.5)	0.555
	N2a	31	16	(30.2)	15	(24.2)	
	N2b	35	12	(22.6)	23	(37.1)	
	N2c	6	3	(5.7)	3	(4.8)	
	N3	26	14	(26.4)	12	(19.4)	
N-category TNM 2017	N1	22	8	(15.1)	14	(22.6)	0.494
	N2	1		–	1	(1.6)	
	N2a	15	9	(17.0)	6	(9.7)	
	N2b	13	7	(13.2)	6	(9.7)	
	N2c	2		–	2	(3.2)	
	N3a	3	2	(3.8)	1	(1.6)	
	N3b	59	27	(50.9)	32	(51.6)	
p16 IHC	positive	10		–	10	(16.1)	**4.60** × **10^−6^**
	negative	13		–	13	(21.0)	
	unknown	92	53	(100)	39	(62.9)	
HR-HPV DNA	positive	10		–	10	(16.1)	**8.92** × **10^−6^**
	negative	12		–	12	(19.4)	
	unknown	93	53	(100)	40	(64.5)	
Tobacco smoking	never	27	13	(24.5)	14	(22.6)	0.405
	former	26	9	(17.0)	17	(27.4)	
	current	62	31	(58.5)	31	(50.0)	
Alcohol	never	24	9	(17.0)	15	(24.2)	0.563
	former	12	5	(9.4)	7	(11.3)	
	current	79	39	(73.6)	40	(64.5)	
Alcohol per day	0	24	9	(17.0)	15	(24.2)	0.431
	1–30 g	36	15	(28.3)	21	(33.9)	
	31–60 g	22	10	(18.9)	12	(19.4)	
	>60 g	33	19	(35.8)	14	(22.6)	

^#^P value from Pearson’s Chi-square (χ^2^) tests; p16 IHC, detection of ≥70% p16^INK4A^ expressing cells in immunohistochemistry (IHC) using CINtec Plus™ kit defines p16-positive NSCCUP; HR-HPV DNA, high-risk human papillomavirus-subtype DNA detected by hybridization using the Inno-LiPA HR-HPV detection kit™. P values from Pearson’s Chi-square tests < 0.05 are in bold.

**Table 2:** Diagnostic procedures and treatment regimens applied to neck squamous cell carcinoma of unknown primary (NSCCUP) patients of cohorts 1 and 2.

Diagnostics and Therapy		Total	Cohort 1 n (%)		Cohort 2 n (%)		*P* value#
PET-CT	no	63	52	(98.1)	11	(17.7)	**6.03** × **10^−18^**
	yes	52	1	(1.9)	51	(82.3)	
Tonsillectomy	yes	48	4	(7.5)	44	(81.0)	**1.21** × **10^−14^**
	no	60	49	(92.5)	11	(17.7)	
	earlier	7		–	7	(11.3)	
First therapy regimen	Op	16	2	(3.8)	14	(22.6)	**2.61** × **10^−4^**
	Op+POCT	3	2	(3.8)	1	(1.6)	
	Op+PORT	43	31	(58.5)	12	(19.4)	
	Op+PORCT	48	17	(32.1)	31	(50.0)	
	pCRT	3	1	(1.9)	2	(3.2)	
	pRT	2		–	2	(3.2)	
Neck dissection (ND)	No	26	18	(34.0)	8	(12.9)	**0.007**
	Yes	89	35	(66.0)	54	(87.1)	
ND	both sides	38	4	(7.5)	34	(54.8)	**4.17** × **10^−7^**
(SND/MRND/RND)	one side	51	31	(58.5)	20	(32.3)	
	none	26	18	(34.0)	8	(12.9)	
Extranodal extension	positive	62	27	(50.9)	35	(56.5)	**2.61** × **10^−5^**
	negative	18	1	(1.9)	17	(27.4)	
	unknown	35	25	(47.2)	10	(16.1)	
Resection margins	R0	76	28	(52.8)	48	(77.4)	**0.005**
	other‡	39	25	(47.2)	14	(22.6)	
Chemotherapy	Cisplatin	22	4	(7.5)	18	(29.0)	**0.010**
	Cisplatin & 5-FU	26	10	(18.9)	16	(25.8)	
	Carboplatin	2	2	(3.8)		–	
	Other	6	4	(7.5)	2	(3.2)	
	None	59	33	(62.3)	26	(41.9)	
Irradiation type	IMRT	47	2	(3.8)	45	(72.6)	**1.37** × **10^−19^**
	Other	49	47	(88.7)	2	(3.2)	
	None	19	4	(7.5)	15	(24.2)	

^#^P value from Pearson’s Chi-square (χ^2^) tests; PET-CT, [^18^F]-FDG-positron emission tomography-computed tomography; Op, surgical resection of neck nodes; POChT, post-operative (adjuvant) chemotherapy; PORT, post-operative (adjuvant) radiotherapy; PORCT, post-operative (adjuvant) radio-chemotherapy; pCRT, primary (definitive) combined radio-chemotherapy; pRT, primary (definitive) radiotherapy; SND/MRND/RND, selective/modified radical/radical neck dissection; 5-FU, 5-fluorouracil; IMRT, intensity-modulated radiation therapy; ‡other summarizes R1, R2 or core biopsies taken prior to pR(C)T. P values from Pearson’s Chi-square tests < 0.05 are in bold.

**Table 3:** Various survival measures for 5-years outcome of neck squamous cell carcinoma of unknown primary (NSCCUP) patients of cohorts 1 and 2.

5 years survival rate		Total	Cohort 1 n (%)		Cohort 2 n (%)		*P* value#
OS	alive	65	24	(45.3)	41	(66.1)	**0.025**
	dead	50	29	(54.7)	21	(33.9)	
TSS	alive or NCRD	86	37	(69.8)	49	(79.0)	0.256
	CRD	29	16	(30.2)	13	(21.0)	
CRD & NCRD	alive	65	24	(45.3)	41	(66.1)	0.072
NCRD CRD		21	13	(24.5)	8	(12.9)	
		29	16	(30.2)	13	(21.0)	
CRD	alive	65	24	(45.3)	41	(66.1)	0.098
	CRD	29	16	(30.2)	13	(21.0)	
NCRD	alive	65	24	(45.3)	41	(66.1)	**0.044**
	NCRD	21	13	(24.5)	8	(12.9)	
DFS	no event	76	33	(62.3)	43	(69.4)	0.423
	event	39	20	(37.7)	19	(30.6)	
PFS	no event	74	32	(60.4)	42	(67.7)	0.411
	event	41	21	(39.6)	20	(32.3)	
Primary	no event	107	47	(88.7)	60	(96.8)	0.089
detected	event	8	6	(11.3)	2	(3.2)	
Nodal control	no event	105	50	(94.3)	55	(88.7)	0.285
	event	10	3	(5.7)	7	(11.3)	
Distant control	no event	104	51	(96.2)	53	(85.5)	0.051
	event	11	2	(3.8)	9	(14.5)	

^#^P value from Pearson’s Chi-square (χ2) tests; ‡ other summarizes R1, R2 or core biopsies taken prior to R(Ch)T; OS, overall survival; TSS, tumor-specific survival; CRD, cancer related death; NCRD, non-cancer related death/death from other cause; DFS, disease-free survival; PFS, progression-free survival; LC, detection of head and neck squamous cell carcinoma primary; NC, nodal control; and DC, distant control. P values from Pearson’s Chi-square tests < 0.05 are in bold.

### CT and PET-CT Imaging

According to clinical guidelines, all patients received a thorax and a head and neck CT scan during staging. In 2006, a PET-CT became available. Beginning in 2007, all patients with clinical diagnosis of NSCCUP were scheduled to receive a [^18^F]-FDG-PET-CT scan (11, 15); however, 11 (17.7%) had no PET-CT imaging before biopsies were taken (**Table 2**).

PET-CTs were analyzed by an experienced board-certified nuclear-medicine physician and a radiologist. Sites of tumor involvement were identified visually by enhanced, non-physiologically [^18^F]-FDG uptake.

### Decision-Making Process in the MDTB

The decision-making process in the MDTB followed ASCO and NCCN guidelines (7, 8) and principles published earlier (12–14, 16–18). Briefly, radiologist and nuclear medicine specialist presented all radiological imaging. The MDTB consisting of head neck and maxillofacial surgeons, a pathologist, an oncologist, a radiation oncologist, and other clinical staff involved in the treatment of head and neck cancer patients discussed results of diagnostic procedures. Considering the general health and comorbidity of the patient the pre-therapeutic MDTB according to guidelines (7, 8) mostly recommended neck dissection as first treatment.

### Histological Examination and HPV Status

All resected specimen underwent pathological examination. Pathological reports were available for all 115 patients. Besides hematoxylin–eosin (HE) staining, molecular analyses of p16 by immunohistochemistry utilizing the CINtec kit were routinely performed since 2013. A sub-cohort of patients participated in a study approved by our Ethics Committee (votes 201-10-12072010 and 202-10-12072010). **Table 1** includes some data respective to HR-HPV published elsewhere (19).

### Treatment Modalities

After ND and obtaining the pathology reporting evident risk factors (especially ECE+), the post-operative MDTB recommended mostly cisplatin-based post-operative chemo-radiotherapy (PORCT) according to guidelines (7, 8, 20). In cohort 2, NSCCUP-P with ND and detection of only unilateral N+ (N2b) without risk factors present (up to 2 N+ <6 cm, no ECE, R0/no incision biopsy) received PORT of 60 Gy ipsilateral and 50 Gy contralateral, independent from ND also of the unaffected site or not. Irradiation after resection of a single node without risk factors (<6 cm, no ECE, R0/no incision biopsy) was unilateral 60 Gy, whereas presence of risk factors for local recurrence (bilateral N+, N2c, or one node ≥6 cm, N3, or ECE+, R1) indicated the need for bilateral irradiation with 64 Gy and concomitant cisplatin 200 mg/m^2^. In cohort 2, according to our standardized protocol and dependent on affected lymph node levels, up to 50 Gy were applied prophylactic to the pharynx (including base of the tongue) and nasopharynx.

In majority, the NSCCUP-P agreed and received MDTB-recommended treatment. Only eight cohort-2 patients disagreed with the recommendation, refused PORT or PORCT, and had solely surgery (**Table 2**).

### Statistical Analysis

Patient characteristics and follow-up data from TDB and OncoFlow® were analyzed in relation to clinical characteristics of patients, risk factors (daily alcohol consumption categorized in 0, ≤30 g, ≤60 g, >60 g; tobacco smoking categorized in ≤ or > 10 pack years; smoking status categorized in never, former, current smoker), diagnostic procedures used, treatment modalities, N and M categories, HPV status. Associations between categorical variables were examined by *Pearson’s Chi-square* test. We calculated overall survival (OS) time from date of diagnosis to date of death (event), or end of follow-up (censored); tumor-specific survival (TSS) time from date of diagnosis to date of cancer-related death (event) censoring other causes of death or end of follow-up. Time to non-cancer related death (NCRD) defined events as death from other cause censoring cancer-related death. Disease-free survival (DFS) measured time-interval from curative R0 resection or last fraction of irradiation to relapse or death (event) censoring patients alive with no sign of disease at last follow-up. We calculated progression-free survival (PFS) time from date of diagnosis to date of relapse or death (event), or end of follow-up without progression (censored). We defined nodal control (NC) as time from NSCCUP diagnosis to relapse focusing on detected disease-positive nodes independent from neck laterality, and measured distant control (DC) as time from NSCCUP diagnosis to distant metastasis detected. We additionally recorded any detection of a primary squamous cell carcinoma in the head and neck region (HNSCC detection). We used receiver-operating characteristic (ROC) curves to find optimum cut-off values for quantitative parameters to binary classify patients. We analyzed survival using the Kaplan–Meier method (21) applying log-rank tests (22) and hazard ratios (*HR*) using Cox proportional hazard models (23) utilizing the conditional logistic regression forward method, and bootstrapping (24) (SPSS version 24, IBM Corporation, Armonk, New York). We considered 2-sided *P <*0.05 significant.

## Results

### Patient Characteristics

The CONSORT diagram (**Figure 1**) shows eligible and excluded patients in cohorts 1 and 2, highlighting increased detection of primaries with standardized clinical workup (*P* = 0.026). The distribution in histologically defined entities and localization of SCC primary lesions detected also differed substantially (*P* = 0.006 and 0.001, respectively). **Table 1** shows patients’ characteristics. Cohorts differed insignificantly regarding sex, history of consuming alcohol or nicotine and age at diagnosis, whereas applied diagnostic and treatment procedures differed significantly (**Tables 1** and **2**).

### PET-CT Analyses

According to PET-CT availability until 2006, 1/53 (1.9%) NSCCUP-P of cohort 1 and 51/62 (82.3%) of cohort 2 underwent [^18^F]-FDG-PET-CT imaging as part of the pre-therapeutic staging (*P* = 6.03 × 10^−18^). All analyzed NSCCUP were detected positive for [^18^F]-FDG enrichment, whereas non-physiologically enhanced uptake by other organs was absent or led to diagnosis of the initially occult primary (**Figure 1** and **Table 2**).

### Tonsillectomy, Neck Dissection and Pathological Examination

According to our in-house standard operating procedure (SOP) defined for diagnostic and treatment of suspect NSCCUP-P in 2007, we exclusively performed surgery after completed staging and imaging and omitted wedge excisions as recommended (7, 8). Surgery then consisted of ND at least of the affected site combined with extended bilateral tonsillectomy, both performed more frequently (*P* = 0.007 and 1.21 × 10^−14^, respectively). This altogether led to improved identification of primary tumors, predominantly cancers of the tongue base and the palatine tonsils (**Figure 1**). Cohorts 1 and 2 differed significantly regarding the depth of clinical examination documented in the pathologic reports; ECE+ and, in case of ECE+, especially R0 resection with >5 mm distance to clear margins were more frequent in cohort 2 (**Table 2**).

### Prevalence of HPV in NSCCUP

As previously shown in a separate analysis of 46/62 NSCCUP-P with tumor and blood samples available (cohort 2), we analyzed HPV DNA, HPV E6*I mRNA and p16^INK4A^ as well as antibodies to HPV proteins in sera (19). We found 9/62 (14.5%) HPV-driven cases defined by positivity for p16^INK4A^ together with presence of HR-HPV DNA and E6*I mRNA. One patient each was negative for either p16^INK4A^ (1/10) or HR-HPV DNA and E6*I RNA (1/10; **Table 1**). Further 3 NSCCUP-P had no FFPE specimen available to analyze p16 and HPV and were defined as HPV-driven based on HR-HPV serostatus (19).

### Treatment Modalities

The treatment of NSCCUP-P differed significantly between cohorts (**Tables 2** and **3**). Bilateral tonsillectomy and ND improved detection of primaries. ND, however, also led to increased detection of ECE in cohort 2.

For a singular SCC-positive node (N1 according to TNM 7^th^ ed. without ECE), the MDTB recommended clinical follow-up, and therefore sole surgery without adjuvant treatment increased in cases without risk factors, whereas, based on risk-adapted decision-making, cisplatin-based Op+PORCT increased. Related to increased cisplatin usage from 14/53 (26.4%) to 34/62 (54.8%), chemotherapies increased overall (*P* = 0.01). After 2006, we predominantly (*P* = 1.37 × 10^−19^) applied intensity-modulated radiotherapy (IMRT). However, irradiation time, fractionation and nominal doses administered by fixed-field radiation (cohort 1) or IMRT (cohort 2) differed not significantly.

### Survival in Cohorts 1 and 2

**Figure 2:**
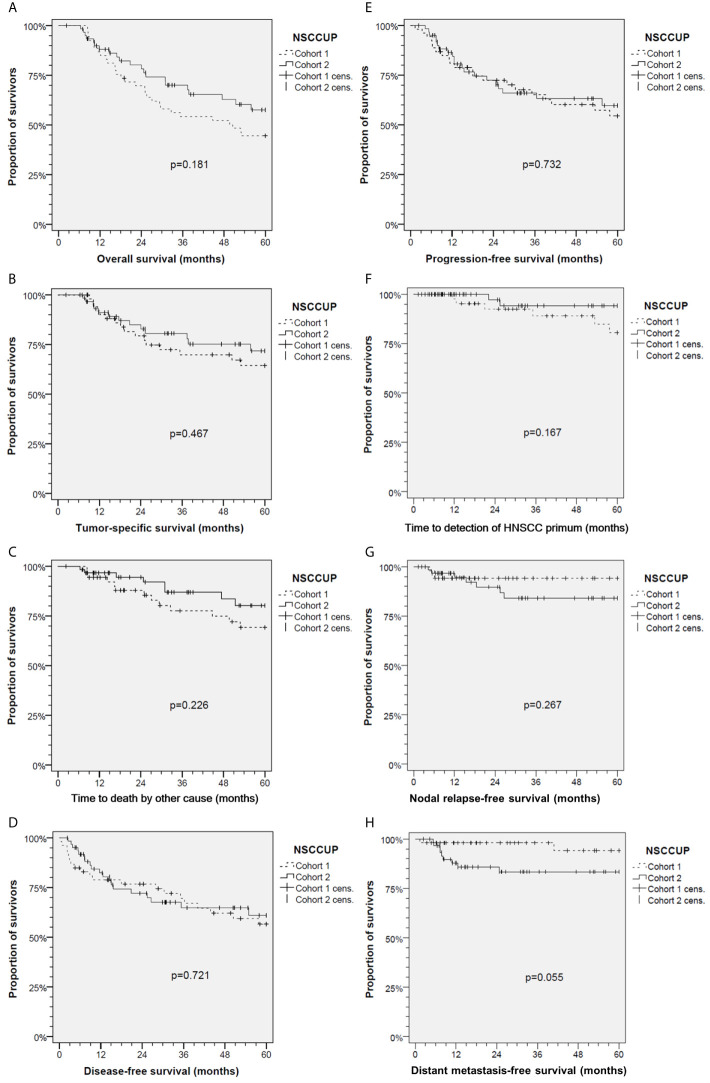
Kaplan–Meier cumulative survival analyses of neck squamous cell carcinoma of unknown primary (NSCCUP) patients of cohorts 1 and 2 for **(A)** overall survival; **(B)** tumor-specific survival; **(C)** survival according to non-cancer death/death from other cause; **(D)** disease-free survival; **(E)** progression-free survival; **(F)** Time to detection of head and neck squamous cell carcinoma primary; **(G)** Nodal relapse-free survival and **(H)** Distant metastasis-free survival. *P* values shown are from 2-sided log-rank tests.

The median follow-up was 37.4 months in the total sample, and 49.6 and 32.9 months in cohorts 1 and 2 (*P* = 0.198). Outcome assessed at 2 years differed only insignificantly. In the total cohorts 1 *versus* 2, 2-years OS was 69.7% (95% CI 57.3–82.1%) *versus* 80.2% (95% CI 69.7–90.8%); 2-years TSS was 79.3% (95% CI 67.8–90.8%) *versus* 84.9% (95% CI 75.2–94.6%); 2-years DFS was 74.4% (95% CI 62.4–86.5%) *versus* 70.3% (95% CI 57.9–82.6%); 2-years loco-regional control 87.1% (95% CI 77.2–96.8%) *versus* 87.2% (95% CI 77.4–97%); 2-years DC was 98.1% (95% CI 94.5–100.0%) *versus* 85.8% (95% CI 76.7–95%). The 5-years OS in cohort 2 was improved (*P* = 0.025); other outcome measures did not demonstrate significant improvements at this time (**Table 3**). Kaplan–Meier plots of cumulative survival (**Figure 2**), however, show improved outcome in cohort 2 but without reaching statistical significance.

### Survival Related to Non-Standardized Versus Standardized Diagnostic Procedures

We analyzed a possible link between non-standardized versus standardized diagnostic procedures. Frequency of PFS and DFS events differed not significantly between cohorts; one patient from cohort 1 had a PFS event related to lung cancer of other histology (no DFS event). No particular diagnostic procedure alone improved outcome (all *P* > 0.2).

### Survival Related to N category, ND and PORCT

The number of neck nodes removed and found positive for disease (N+) defined the N category linked to different outcome (**Table S1**). According to Kaplan–Meier curves, NSCCUP-P of cohorts 1 and 2 undergoing ND had identical OS and TSS.

**Figure 3:**
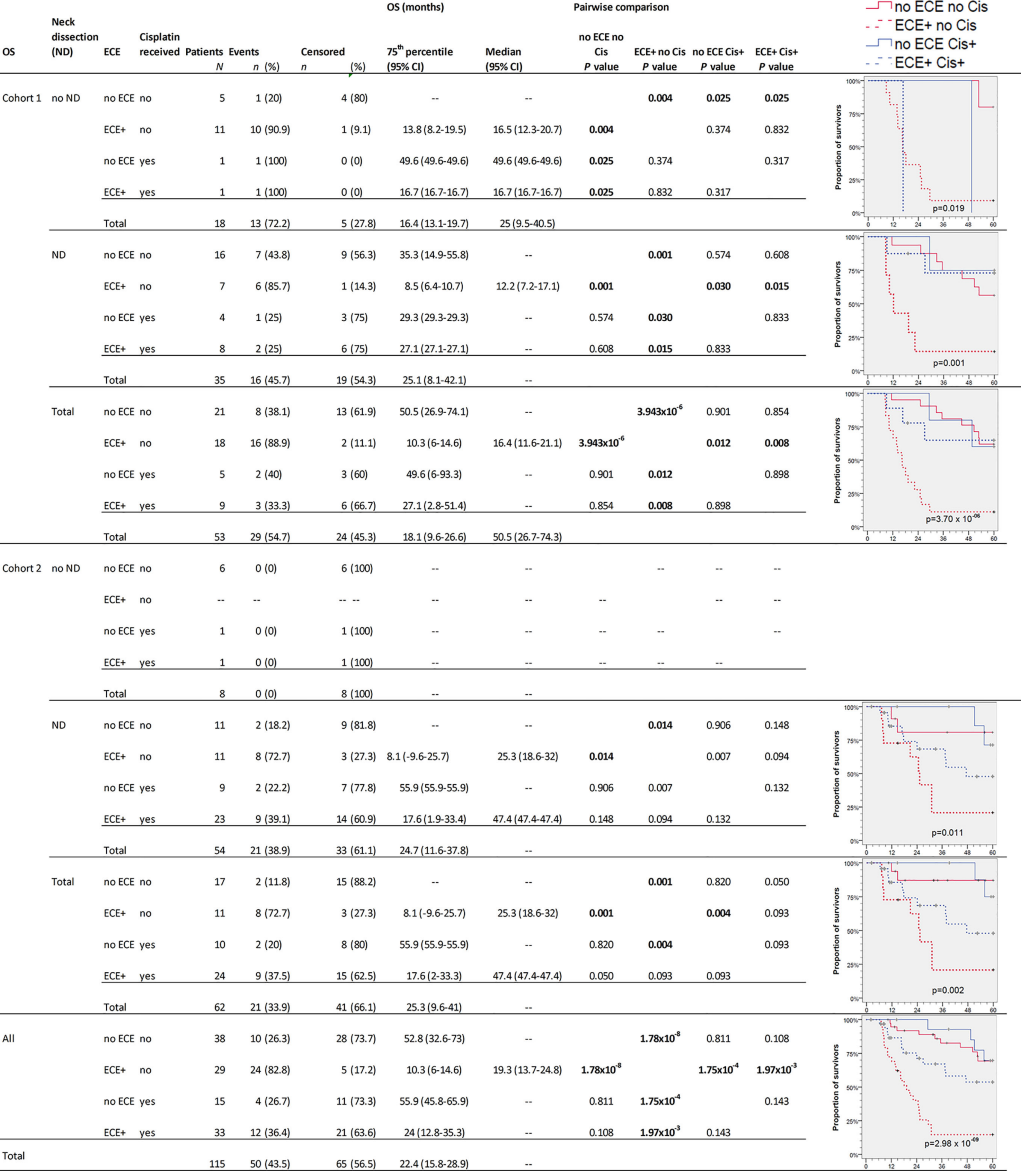
Kaplan–Meier cumulative survival (KM) plots for overall survival (OS) of squamous cell carcinoma of unknown primary patients (NSCCUP-P) demonstrate the improved outcome achieved by standardized treatment including neck dissection (ND) and cisplatin-based radio-chemotherapy (Cis+) for NSCCUP-P with extracapsular extension of neck nodes (ECE). Besides KM plots, the numbers of patients and events are shown with 75th percentile and median along with their respective 95% confidence interval (95% CI) together with *P*-values from pairwise comparisons in various strata combining ND, ECE and Cis in cohorts 1 and 2 as well as for all NSCCUP-P.

**Figure 4:**
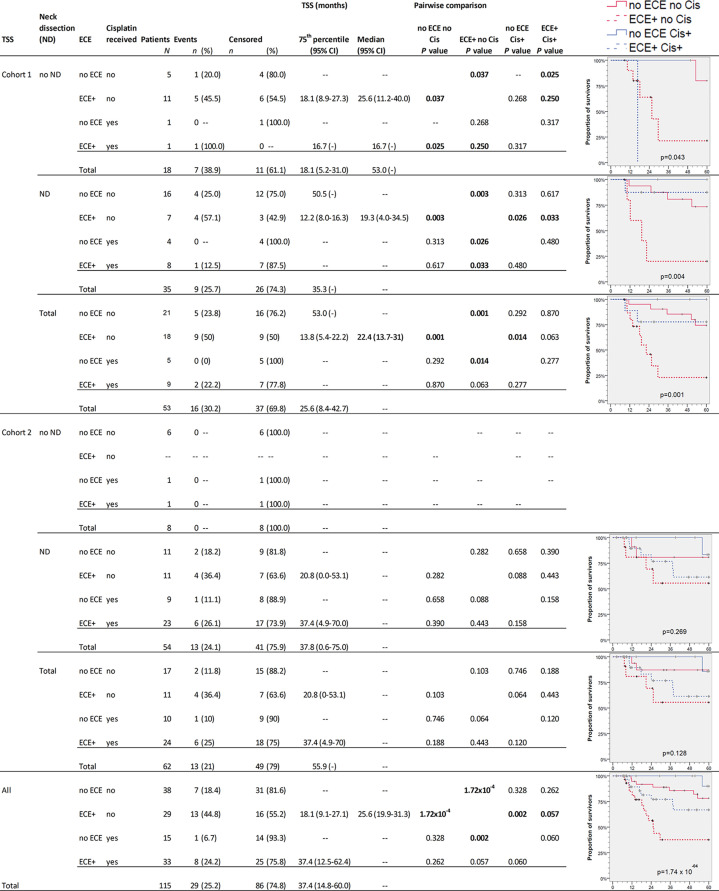
Kaplan–Meier cumulative survival (KM) plots for tumor-specific survival (TSS) of squamous cell carcinoma of unknown primary patients (NSCCUP-P) demonstrate the improved outcome achieved by standardized treatment including neck dissection (ND) and cisplatin-based radio-chemotherapy (Cis+) for NSCCUP-P with extracapsular extension of neck nodes (ECE). Besides KM plots, the numbers of patients and events are shown with 75th percentile and median along with their respective 95% confidence interval (95% CI) together with *P*-values from pairwise comparisons in various strata combining ND, ECE and Cis in cohorts 1 and 2 as well as for all NSCCUP-P.

The survival in NSCCUP without ND differed significantly regarding TSS (7/18 CRD), death by other cause (6/18 NCRD) and OS (13/18 deaths in total) in cohort 1 versus 0/8 in cohort 2 (*P* = 0.076, 0.117 and 0.018, respectively, in log-rank tests). The highest differences in OS and TSS relate to ND, detection of ECE, and risk factor adapted use of cisplatin-based regimens (**Figures 3** and **4**).

We detected a significantly improved outcome (*P* = 2.98 × 10^−9^ and 1.74 × 10^−4^ for OS and TSS, respectively) of patients receiving cisplatin-based Op+PORCT. Those patients who had ECE but did not receive cisplatin-based Op+PORCT experienced worst outcome with median OS and TSS of 19.3 (95% CI 13.7, 24.8) and 25.6 (95% CI 19.9, 31.3) months only. Significant best outcome had patients without ECE (**Figures 3** and **4**). Comparing PORT and cisplatin-based PORCT after ND of NSCCUP without ECE of both cohorts combined, we detected numerical improved TSS according to 6 cancer-related deaths in 27 NSCCUP-P after PORT (22.2%) *versus* 1 cancer-related death (7.7%) in 13 NSCCUP-P receiving PORCT (*P* = 0.257). However, the corresponding Kaplan–Meier plots and log-rank test confirmed that the TSS difference between PORT and PORCT was not significant (*P* = 0.266).

### Survival Related to HPV

As shown previously, HPV-related NSCCUP-P demonstrated significant superior OS and PFS in univariate analyses (19) therefore they are not presented here again.

### Multivariate Cox Regression Models for Outcome

**Figure 5:**
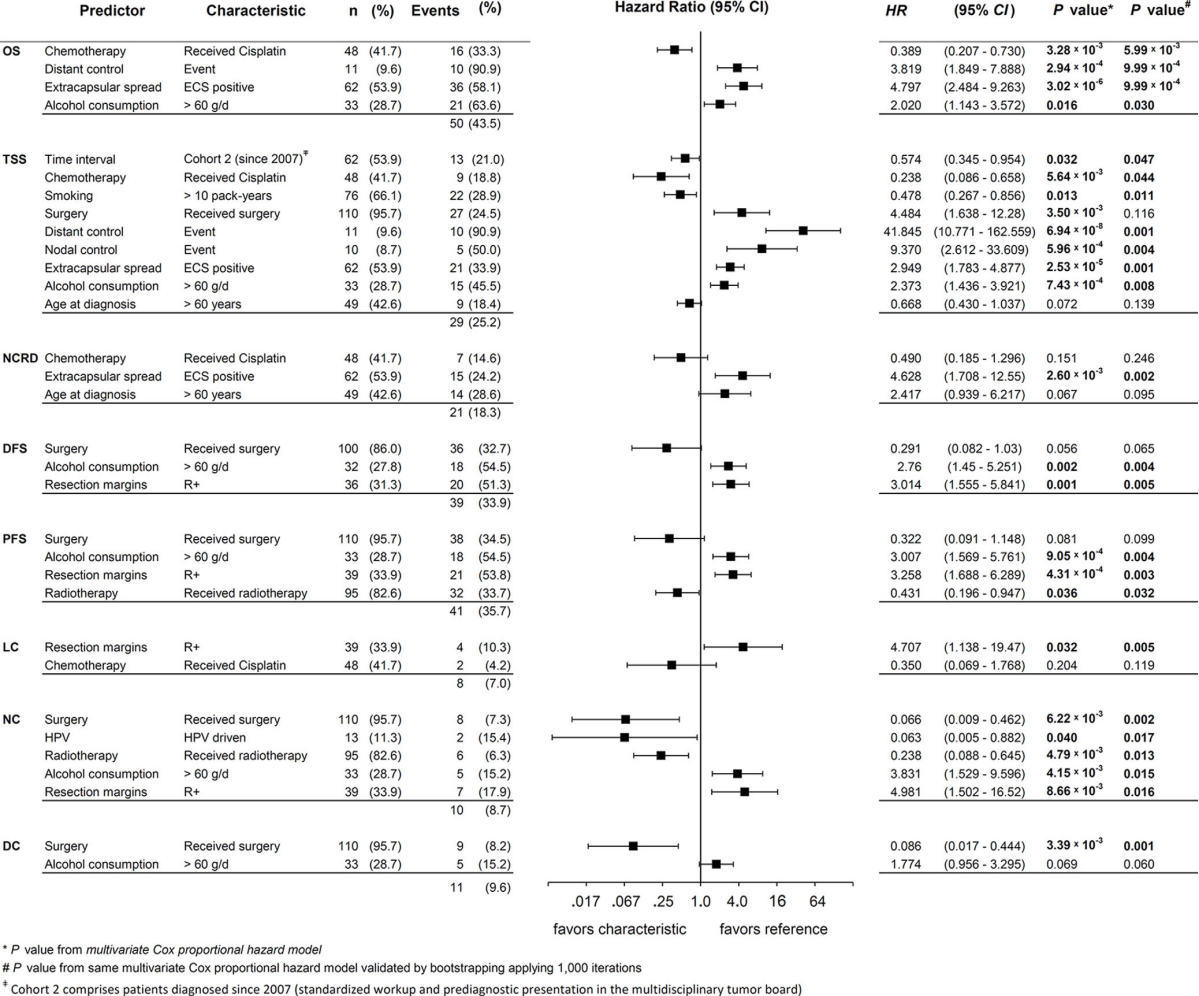
Forest plots for hazard ratio (*HR*) and 2-sided 95% confidence interval (95% CI) from multivariate Cox proportional hazard models for various outcome measures and survival of neck squamous cell carcinoma of unknown primary (NSCCUP) patients built using the stepwise forward likelihood ratio method. Events represents the numbers of events found for the individual predictor and (%) the percentage of patients experiencing the event among those with the characteristic, and *HR*, 2-sided 95% CI, and 2-sided *P* value* the outcome attributable to this characteristic according to the final model for the particular measure. *P* values^#^ are 2-sided *P* values from internal validation using bootstrapping applying 1,000 iterations. *P* values of independent predictors < 0.05 are in bold.

**Figure 5** shows forest plots, *HR*, 95% CI, and the corresponding *P* values of covariates in the multivariate *Cox* proportional hazard model achieving highest significance for the respective outcome measure. As recommended (24), we show *p* values from bootstrapping utilizing 1,000 iterations for independent predictors (*Pi*). This internal validation revealed significance of all models and confirmed all *Pi* being significant with only one exception: The (adverse) effect of surgery on TSS that lost significance applying the bootstrap. Thus, we identified the appearance of surgery as designated *Pi* as an artifact of the statistical model because surgery improves NC and DC. On the opposite, both types of event, NC events (nodal failure) and DC events (distant failure) led to impaired TSS.

Consequently, nodal and distant failure are linked to treatment without surgery (compare the opposite direction of effects in multivariate analyses including DC and NC exerted by surgery on TSS *versus* the effects of surgery on DC and NC; **Figure 5**). Related to confounding by factors also linked to improved outcome in the total NSCCUP cohort, multivariate analyses did not confirm any significant impact of HPV-driven disease on survival. With NC being the only exception, the close correlation of HPV status and predominant *Pi* demonstrates that HPV-relatedness is rather a confounder linked to many independent predictors of improved survival without itself being an independent predictor of improved outcome (**Figure 5**).

We identified the standardized diagnostic workup and decision-making for surgery followed by risk-factor adapted adjuvant therapy applied to NSCCUP-P since 2007 as a significant *Pi* for improved TSS.

## Discussion

Our retrospective study demonstrates improved outcome in NSCCUP achieved through evidence-based decision-making by a MDTB for the probably best treatment option for the individual NSCCUP-P based on standardized diagnostic workup including [^18^F]-FDG-PET-CT imaging, bilateral tonsillectomy and neck dissection. Our MDTB comprises all professions involved in the clinical workup of head and neck cancer patients (12). Pre- and post-surgery presentation of each NSCCUP case in the MDTB along with SOPs in diagnostics and treatment mirror the recently published guidelines (7, 8).

To overcome variable preoperative workup, we implemented a standardized diagnostic approach including contrast-enhanced CT and PET-CT imaging followed by EUA with random biopsies taken from the base of the tongue or lingual tonsillectomy, biopsies from the epipharynx followed by bilateral tonsillectomy and ND. The increased detection of occult tumors demonstrates that this standardized workup is effective. The diagnosis of occult tumors was facilitated (**Figure 1**), and the number of primary SCC detected at various head and neck sites increased substantially (*P* = 0.001). Shortly after establishing comprehensive use of PET-CT for CUP patients in our clinic in 2007, the prospective DAHANCA-13-study showed changes in therapeutic strategy in about 25% of NSCCUP-P after diagnosis of the primary tumor using PET-CT in diagnostic workup und also recommended use of FDG-PET for diagnostic processes in NSCCUP-P (25). Our results are in line with their findings (**Figure 1**). The high rate of subclinical (occult) primary lesions detected by PET-CT and EUA confirms recent results from a meta-analysis demonstrating the highly increased probability to detect small lesions given combined utilization of these modern approaches (26). Mostly, the primary tumors identified in our cohort were located in the base of tongue or palatine tonsils, in line with recent data (27, 28). At time of NSCCUP diagnosis and more than 6 months thereafter a total of six (five within 60 months) occult HNSCC tumors were detected in our cohort 1 but only two in cohort 2 (compare **Table 1** and **Figure 2F**).

Besides improved outcome in the oropharyngeal cancers presenting initially as NSCCUP (data not shown), we provide evidence that a standardized diagnostic workup followed by risk-adapted treatment described improves the outcome even in definitive NSCCUP-P without detection of primary lesions (**Table 1** and **Figures 2**–**5**). The evidence-based decision-making for further diagnostic and the standardized treatment planning for CRT, Op + PORT or risk-adapted cisplatin-based Op + PORCT improve outcome as demonstrated in oropharyngeal cancer (29). The diagnostic step carrying a substantial therapeutic effect improving survival was ND. Kaplan–Meier curves and 75th percentiles highlight comparable OS (27.1 *versus* 24.7 months) and TSS (35.3 v*ersus* 37.4 months) of NSCCUP-P undergoing ND in both cohorts (**Figures 3** **,** **4** and **Table S1**). In contrast, OS in NSCCUP-P without ND differed significantly with 13/18 (72%) deaths in cohort 1 (CRD: 7/18, NCRD: 6/18) *versus* 0/8 in cohort 2 (*P* = 0.076, 0.117, and 0.018, respectively). None of the 18 cohort 1-patients without ND underwent tonsillectomy; 8/18 (44.5%) experienced wedge excision and 10/18 (55.5%) node extirpation only. The eight NSCCUP-P of cohort 2 without ND had extended tonsillectomy in 7/8 (87.5%) and extirpation of their singular neck node in 6/8 (75%) cases. Those two NSCCUP-P of cohort 2 without ND or node extirpation received curative IMRT applying cisplatin-based CRT and definitive RT, respectively. The latter age-79 male with high comorbidity is disease-free now for more than 38 months.

Strikingly, N categories according to TNM 2017 (8th edition) proofed to be superior regarding prognostication of survival especially when considering results of p16 immunohistochemistry (leading to down-staging of p16+ NSCCUP) and ECE (up-staging of p16-negative NSCCUP with ECE+). Increased ECE detection, however, was dependent on a multitude of effective changes facilitating in-depth pathological examination in NSCCUP-P of cohort 2.

Given the heterogeneity of NSCCUP and the small case numbers compared, the general survival benefit for the total cohort 2 was below statistical significance (according to log-rank tests) (26).

However, the subgroup analyses presented for OS (**Figure 3**) and TSS (**Figure 4**) demonstrate that multimodal therapy combining upfront surgery (ND and tonsillectomy) followed by cisplatin-based PORCT are able to overcome the prior significant worse outcome of ECE+ NSCCUP and hence is strongly recommended. ECE is discussed in various retrospective studies whether (30) or not (31) being a prognostic factor for poorer survival. While demonstrating the negative impact of ECE on OS, TSS, and NCRD and being a significant *Pi* for impaired survival even after internal validation by bootstrapping, we showed importance of considering ECE in decision making for risk-factor adapted therapy and favoring cisplatin-based chemotherapy to overcome the increased risk of dying (**Figures 3** and **4**). In cohort 2, the increased utilization of cisplatin-based PORCT led to improved survival and reduced the difference between NSCCUP-P with and without ECE. Whenever cisplatin is used in risk-adapted adjuvant treatment for ECE+ detected in resected nodes, differences diminished to insignificance. Therefore it is no surprise that NSCCUP-P of cohort 1 undergoing ND followed by cisplatin-based PORCT had the same survival as those in cohort 2 (**Figures 3** and **4**). This finding hence is compatible with a significant negative impact of ECE+ in a cohort with low frequent (29%) use of PORCT (30) comparable to our cohort 1 (26.4%) that also showed significant impaired survival in ECE+ patients, whereas use of cisplatin-based PORCT in cohort 2 was 54.8% and linked to improved survival. This benefit in TSS and OS in patients receiving cisplatin-based PORCT that could not be shown in rather small NSCCUP-P studies before (30, 32) confirms findings in advanced head and neck cancers (16, 17, 20). Whereas ND followed by cisplatin-based PORCT should be recommended to NSCCUP-P with ECE+, it is not yet completely clear if the numerical benefits regarding OS and especially TSS in NSCCUP without ECE seen in our study outweigh the potential harms from cisplatin-based PORCT. A randomized controlled trial addressing this question by analyzing outcome including health-related quality of life would allow to obtain the required evidence.

In multivariate analyses, the most stable covariates affecting various survival measures were alcohol consumption >60 g/d, ECE+ and positive resection margins (R+), all consistently associated with significant impaired survival, contrary to surgical resection, radiotherapy and cisplatin-based chemotherapy. No differences between use of IMRT or fixed field and applied as adjuvant or definitive irradiation were noticed. Age >60 at diagnosis reduced the risk of dying from cancer (*HR* 0.67, 95% CI 0.43, 1.04) but increased the competing risk regarding death from other causes (*HR* 2.42, 95% CI 0.94, 6.22), and age consequently failed to exert a significant impact on OS.

Our study has limitations. All analyses in NSCCUP including ours suffer from low case numbers especially in a single clinic demanding collection over years. Meanwhile, some changes in clinical practice may have occurred, and these changes may not be limited to those noticed, e.g. cisplatin-based PORCT already introduced in 2004. However, the treatment in cohort 2 is in line with recommendations according to guidelines and clinical evidence (7, 8, 25–28, 30–33). Some analyses suffer from impossibility to obtain pathologic data not included in the available reports, and molecular data including p16 status is missing especially in cohort 1. In addition, Epstein–Barr virus (EBV) was not assessed, as Germany is a low-endemic EBV and nasopharynx cancer region (34) and as EBV included in TNM staging of NSCCUP introduced in 2018 in the 8th TNM edition (35). Unfortunately, no FFPE blocks from cohort 1 were available for EBV detection. Because of the retrospective character of our analyses, and despite great effort to obtain data from printed documentation regarding risk factors, some missing data remained. This could have led to the impossibility in demonstrating any survival benefit of HPV-related NSCCUP in multivariate analyses as missing FFPE blocks in cohort 1 reduced the power to detect such differences substantially.

Strengths of the study are consistency respective to the studied population treated at a single center. According to the latest review, our monocentric study comprising 115 definitive NSCCUP-P out of 272 patients with initially occult primary lesion (**Figure 1**) is the largest single-institution cohort of NSCCUP-P analyzed so far regarding diagnostic procedures, decision-making, treatment regimens and outcome not limited to survival (26, 33). A strength is the internal validation of *Pi* by bootstrapping (24). Paying attention to the heterogeneity of NSCCUP-P, we present evidence for successful treatment stratification possibilities.

## Conclusions

The sound approach recommended by ASCO and NCCN (7, 8) and implemented into our clinical routine along establishing of the MDTB yielded positive effects by choosing the right treatment for the right NSCCUP-P approaching more often the ultimate goal providing the right treatment for the right patient at the right time, all the time (36). ND and cisplatin-based PORCT, whenever advised due to ECE+ or bilateral malignancy-positive neck nodes, improve outcome in NSCCUP-P.

## Data Availability Statement

The raw data supporting the conclusions of this article will be made available by the authors, without undue reservation.

## Ethics Statement

The studies involving human participants were reviewed and approved by Ethics Committee of the University Leipzig (votes 201-10-12072010 and 202-10-12072010). The patients/participants provided their written informed consent to participate in this study.

## Author Contributions

Conceptualization, GW. Methodology, GW. Validation, GW, MW, and TW. Formal analysis, GW, MW, TW, SW, and VZ. Investigation, GW, MW, RK, and TG. Resources, GW, TK, RK, FL, AD, SW, and VZ. Data curation, GW, MW, TW, and SF. Writing—original draft preparation, GW and MW. Writing—review and editing, all authors. Visualization, GW and MW. Supervision, GW, SW, AD, and FL. Project administration, GW. Funding acquisition, GW and AD. All authors contributed to the article and approved the submitted version.

## Funding

The study was partly supported by the grants LIFE-006 B7 and LIFE-007 D9 of the Leipzig Research Center for Civilization Diseases (LIFE) funded by the European Union, the European Fund for Regional Development (EFRE), and the Free State of Saxony. The funding sources did not influence the design of the study, collection, interpretation and analysis of the data, the preparation of this report, or the decision to publish. The authors received support through covering the costs of open access publishing from the German Research Foundation (DFG) and the Open Science Project of the University of Leipzig within the program Open Science Publishing.

## Conflict of Interest

The authors declare that the research was conducted in the absence of any commercial or financial relationships that could be construed as a potential conflict of interest.
