# Supplementary material for: Standardized Diagnostics Including PET-CT Imaging, Bilateral Tonsillectomy and Neck Dissection Followed by Risk-Adapted Post-Operative Treatment Favoring Radio-Chemotherapy Improve Survival of Neck Squamous Cell Carcinoma of Unknown Primary Patients
Source: Front Oncol. 2021 May 7;11:682088. doi: 10.3389/fonc.2021.682088 (PMC8138574; doi:10.3389/fonc.2021.682088)
Supplement: Supplementary file 1 [file DataSheet_1.pdf]

Table S1. Results from Kaplan-Meier cumulative survival plots for median and 75th percentile survival times and p values from log-rank tests as well as hazard ratios (HR) and 95% confidence intervals (CI 95%) in univariate Cox proportional hazard models for overall (OS) and tumor-specific survival (TSS) of various covariates demonstrating an impact on survival of NSCCUP patients according to multivariate Cox proportional hazard models. *P* values below 0.05 are in bold.

**Covariates and Characteristics**		**median (months)**	**75th percentile (months)**	$P_{\text{log rank}}$	$HR_{\text{Cox univariate}}$ **(CI 95%)**	$P_{\text{Cox univariate}}$
**Overall survival**						
Cohort	1	50.5	18.1	0.181	1.464	0.184
	2	-	25.3		(0.835-2.568)	
Age	>60	51.4	25.0	0.412	1.261	0.413
(years)	≤60	-	22.4		(0.723-2.203)	
Smoking	>10	49.6	19.3	0.054	1.876	0.058
(pack years)	≤10	-	35.3		(0.978-3.584)	
Alcohol	>60	25.1	11.3	$\mathbf{3.71 \times 10^{-3}}$	2.252	$\mathbf{4.74 \times 10^{-3}}$
(g/day)	others		29.3		(1.282-3.953)	
HPV16-related	yes	-	-	$\mathbf{2.75 \times 10^{-3}}$	26.06	0.057
	no	50.5	19.3		(0.90-753.3)	
N categories	N3	31.0	16.5	0.152	1.582	0.156
	others	-	24.7		(0.840-2.976)	
N categories	N1	-	53.0	0.086	0.453	0.094

	others	52.8	18.1		(0.180-1.144)	
Neck dissection	no ND	49.6	16.7	0.318	1.380	0.320
	ND	-	24.7		(0.732-2.602)	
Tonsillectomy	no	50.5	16.7	0.201	1.206	0.320
	yes	-	25.3		(0.903-1.610)	
Surgery	yes	-	22.4	0.763	0.804	0.763
	no	55.9	18.1		(0.195-3.321)	
R status	R0	-	37.4	**8.17×10^{-6}**	0.296	**2.54×10^{-5}**
	R+	22.4	12.2		(0.168-0.522)	
ECE	ECE+	25.3	14.2	**6.35×10^{-6}**	3.817	**2.52×10^{-5}**
	no	-	52.8		(2.045-7.092)	
Cisplatin	yes	-	37.4	0.111	0.620	0.115
	no	50.5	18.1		(0.342-1.123)	
Radiotherapy	yes	-	22.4	0.964	0.983	0.964
	no	53.0	25.6		(0.478-2.024)	
PFS	relapse	25.1	12.2	**7.51×10^{-7}**	3.831	**3.99×10^{-6}**
	yes	-	47.4		(2.165-6.803)	
Primary detected	yes	29.6	22.4	0.980	1.013	0.980
	no	-	24.7		(0.364-2.817)	
Nodal control	relapse	37.8	9.0	0.240	1.658	0.603
	NC	-	24.7		(0.707-3.891)	

Locoregional control	relapse	37.8	19.3	0.236	1.517	0.239
	LRC	-	25.0		(0.758-3.03)	
Distant control	relapse	17.6	10.4	**6.83×10^{-6}**	4.464	**3.84×10^{-5}**
	DC	-	25.6		(2.193-9.091)	
Other cancer entity	yes	50.5	20.8	0.639	1.276	0.640
	no	-	24.0		(0.459-3.546)	
**Tumor-specific survival**						
Cohort	1	-	25.6	0.467	1.311	0.469
	2	-	55.9		(0.630-2.726)	
Age	>60	-	-	0.304	0.664	0.307
(years)	≤60	-	25.3		(0.302-1.458)	
Smoking	>10	-	25.3	0.158	1.828	0.165
(pack years)	≤10	-	-		(0.781-4.292)	
Alcohol	>60	50.5	12.2	**6.09×10^{-4}**	3.333	**1.23×10^{-3}**
(g/day)	others	-	-		(1.605-6.944)	
HPV16-related	yes	-	-	**0.024**	25.95	0.151
	no	-	25.6		(0.31-2211.29	
N categories	N3	-	35.3	0.886	1.073	0.886
	others	-	37.5		(0.409-2.816)	

N categories	N1	-	-	0.221	0.481	0.232
	others	-	25.6		(0.145-1.595)	
Neck dissection	no ND	-	29.6	0.625	1.237	0.625
	ND	-	37.8		(0.527-2.905)	
Tonsillectomy	no	-	25.6	0.245	1.252	0.249
	yes	-	55.9		(0.854-1.837)	
Surgery	yes	-	35.3	0.301	0.475	0.312
	no	55.9	18.1		(0.112-2.008)	
R status	R0	-	-	**3.63×10^{-4}**	0.278	**8.27×10^{-4}**
	R+	29.6	17.6		(0132-0.589)	
ECE	ECE+	-	19.3	**7.08×10^{-4}**	3.759	**1.56×10^{-3}**
	no	-	-		(1.656-8.547)	
Cisplatin	yes	-	-	0.198	0.600	0.203
	no	-	25.3		(0.273-1.318)	
Radiotherapy	yes	-	35.3	0.948	1.032	0.948
	no	-	37.4		(0.394-2.71)	
PFS	relapse	25.1	13.8	**5.23×10^{-17}**	257.1	**3.52×10^{-3}**
	yes	-	-		(6.2-10675.8)	
Primary detected	yes	29.6	22.4	0.265	1.808	0.272
	no	-	37.8		(0.629-5.208)	
Nodal control	relapse	55.9	16.7	**0.047**	2.564	0.056
	NC	-	37.4		(0.977-6.711)	

Locoregional control	relapse	37.8	19.3	**0.010**	2.688	**0.014**
	LRC	-	53.0		(1.224-5.917)	
Distant control	relapse	17.6	10.4	**2.76×10^{-11}**	9.434	**3.76×10^{-8}**
	DC	-	37.4		(4.237-20.83)	
Other cancer entity	yes	50.5	20.8	0.116	2.278	0.126
	no	-	37.8		(0.792-6.536)	
